# Correction: Quintero-Espinosa et al. LRRK2 Knockout Confers Resistance in HEK-293 Cells to Rotenone-Induced Oxidative Stress, Mitochondrial Damage, and Apoptosis. *Int. J. Mol. Sci.* 2023, *24*, 10474

**DOI:** 10.3390/ijms27167409

**Published:** 2026-08-19

**Authors:** Diana Alejandra Quintero-Espinosa, Sabina Sanchez-Hernandez, Carlos Velez-Pardo, Francisco Martin, Marlene Jimenez-Del-Rio

**Affiliations:** 1 Neuroscience Research Group, Institute of Medical Research, Faculty of Medicine, University of Antioquia, University Research Headquarters, Calle 62#52-59, Building 1, Laboratory 411/412, Medellin 050010, Colombia; dalejandra.quintero@udea.edu.co (D.A.Q.-E.); calberto.velez@udea.edu.co (C.V.-P.); 2 Genomic Medicine Department, Centre for Genomics and Oncological Research (GENYO), Pfizer-University of Granada-Andalusian Regional Government, Parque Tecnólogico Ciencias de la Salud, Av. de la Ilustración 114, 18016 Granada, Spain; 3 Biochemistry and Molecular Biology 3 and Immunology Department, Faculty of Medicine, University of Granada, Avda. de la Investigacion 11, 18071 Granada, Spain

In the original publication [1], there was a mistake in Figure 5L,P,T as published. During the final assembly of the multichannel immunofluorescence composite figures, the images intended to represent PRKN staining in LRRK2 knockout (KO) cells were inadvertently replaced with images corresponding to a different fluorescence channel used to assess total LRRK2 expression in wild-type (WT) cells, resulting in duplication with Figure 1D,F,H. The corrected Figure 5L,P,T appears below. The authors state that the scientific conclusions are unaffected. This correction was approved by the Academic Editor. The original publication has also been updated.

## Figures and Tables

**Figure 5:**
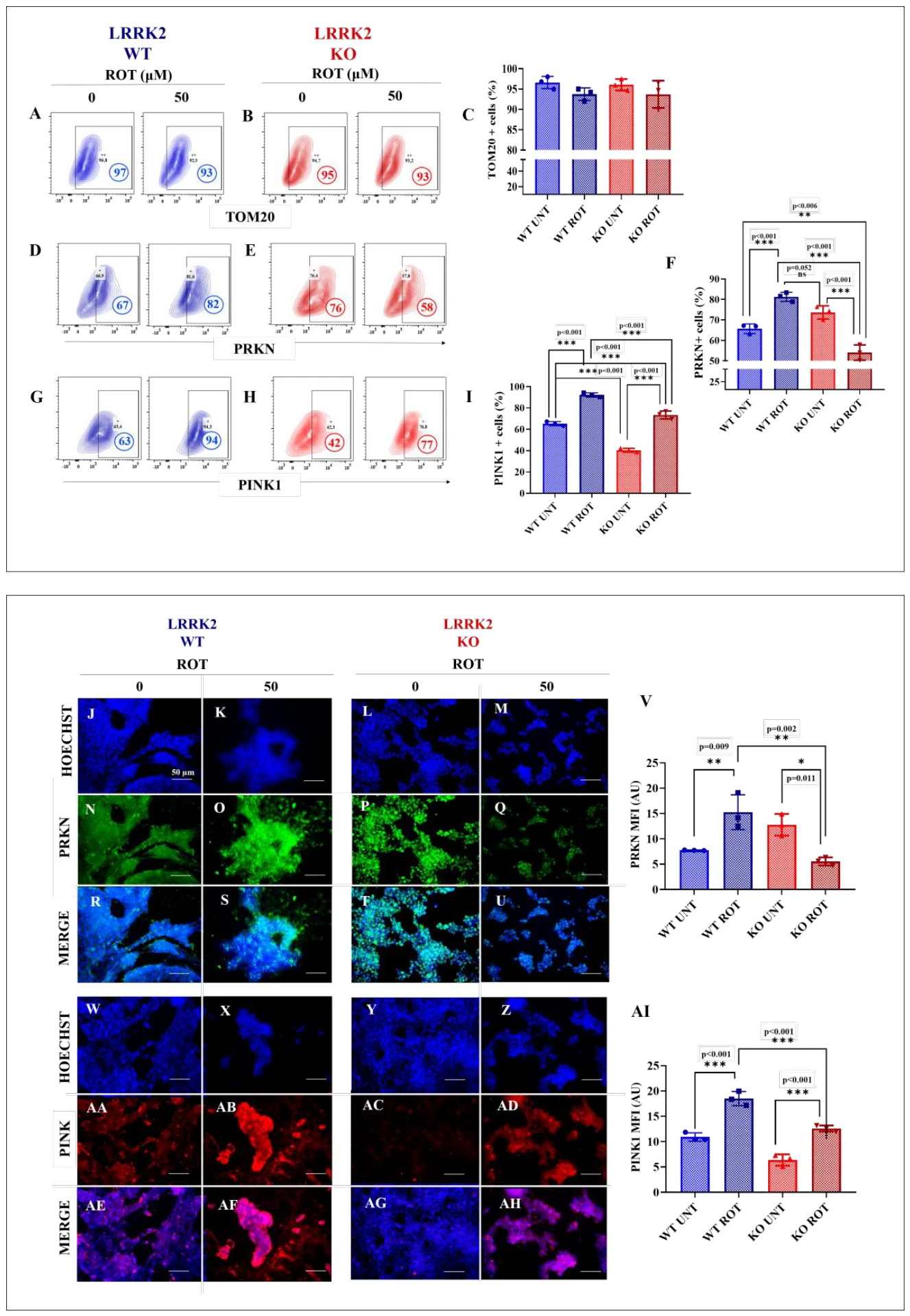
LRRK2 KO upregulates PINK1 but not PRKN under ROT stimuli. (**A**,**B**) Representative flow cytometry contour plots show HEK-293 LRRK2 WT and LRRK2 KO cells untreated or treated with ROT (50 µM) labeled with primary antibodies against TOM20. (**C**) Percentage of TOM20. (**D**,**E**) Representative flow cytometry contour plots show LRRK2 WT and LRRK2 KO cells untreated or treated with ROT (50 µM) labeled with primary antibodies against PRKN. (**F**) Percentage of PRKN. (**G**,**H**) Representative flow cytometry contour plots show LRRK2 WT and LRRK2 KO cells untreated or treated with ROT (50 µM) labeled with primary antibodies against PINK1. (**I**) Percentage of PINK1. The data are presented as the mean ± SD of three independent experiments (n = 3). One-way ANOVA followed by Tukey’s test: Statistically significant differences when ** *p* < 0.01, and *** *p* < 0.001; ns: no significance. In addition, HEK-293 WT LRRK2 cells and KO cells were stained Hoechst (**J**–**M**,**W**–**Z**), primary antibodies against PRKN (**N**–**Q**), and merged (**R**–**U**), PINK1 (**AA**–**AD**), and merged (**AE**–**AH**). Positive blue fluorescence reflects nuclei, positive green fluorescence reflects the cytoplasmic presence of PRKN protein, and positive red fluorescence reflects the presence of PINK1 protein. (**V**,**AI**) Quantification of the PRKN and PINK1 mean fluorescence intensity (MFI) in LRRK2 WT and KO cells. The figures represent one out of three independent experiments, followed by Tukey’s test: Statistically significant differences when * *p* < 0.05, ** *p* < 0.01, and *** *p* < 0.001. Image magnification, 200×.
