# Supplementary material for: LRRK2 Knockout Confers Resistance in HEK-293 Cells to Rotenone-Induced Oxidative Stress, Mitochondrial Damage, and Apoptosis
Source: Int J Mol Sci. 2023 Jun 22;24(13):10474. doi: 10.3390/ijms241310474 (PMC10341561; doi:10.3390/ijms241310474)
Supplement: Supplementary file 1 [file ijms-24-10474-s001.zip › ijms-2374808-supplementary.pdf]

Supplementary Material

Supplementary Fig S1(A). LRRK2 gene edition by CRISPR/Cas9 method in HEK-293 cells. Figure shows three sgRNAs targeting the start codon (ATG) exon 1 of LRRK2 (e.g., 1. Fw sgRNA (gL1), 2. Rv sgRNA (gL2), and 3. Rv sgRNA (gL3), *green* color), with their respective protospacer adjacent motif (PAM, *red* color), and Cas9 cleavage site (*blue* arrowheads) designed using CRISPOR software. **(B). PCR amplicons**. Figure shows PCR amplicons from WT and edited LRRK2 fragments obtained by LRRK2-specific primers (Suppl. Table S1).

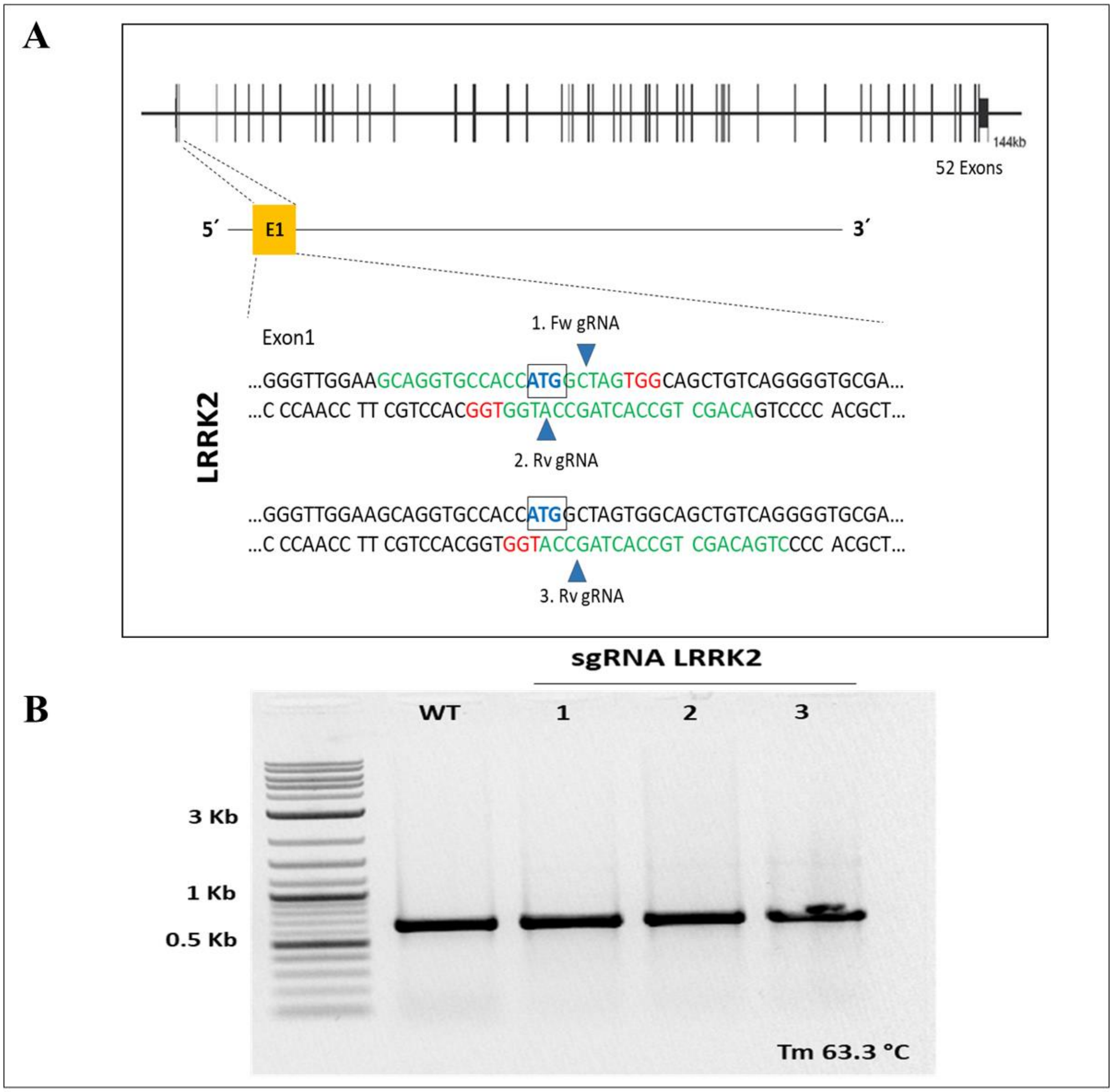

Supplementary Figure S2. Knockout confirmation of LRRK2 using Synthego's Inference of CRISPR Edits (ICE) tool and Flow cytometry. HEK293 cells treated with CRISPR Cas9-RNP containing guides for LRRK2 (gL1, gL2, gL3) to generate knockout (KO) cell lines. **(A-C)** Sanger sequence showing edited and wild-type (control HEK-293 WT LRRK2) sequences in the region around the gRNA LRRK2 gL1, gL2, and gL3 guide sequences. Sequence base calls from both the control (HEK-293 WT LRRK2) and edited.ab1 files, which contained mixed base calls, were illustrated. The horizontal, black, underlined region represents the guide sequence. The horizontal, red, underline is the PAM site. The vertical black dotted line represents the actual cut site. Cutting and error-prone repair usually result in mixed sequencing bases after the cut in the gL2 and gL3 target regions. **(D-F)** The discordance plot details the level of alignment per base between the HEK293 LRRK2 WT (control) and the edited HEK293 LRRK2 KO in the inference window (the region around the cute site). The average amount of signal that disagrees with the reference sequence derived from the control trace file is shown. On the plot, the green line and the orange line should be close together before the cut site, with a typical CRISPR edit resulting in a jump in the discordance near the cut site and continuing to remain far apart after the cut site (representing a high level of sequence discordance). **(G-I)** Indel plot displays the inferred distribution of indels in the HEK293 edited population at gL2 and gL3 target sites. Each bar of the Indel plot shows the size of the insertion or deletion (+ or - 1 or more nucleotides), along with the percentage of genomes that contain it. **(J-L)** Flow cytometry analysis of LRRK2 expression in HEK293 edited L1, L2 and L3 clones (in *red*) in comparison with control (HEK-293 WT in *blue*).

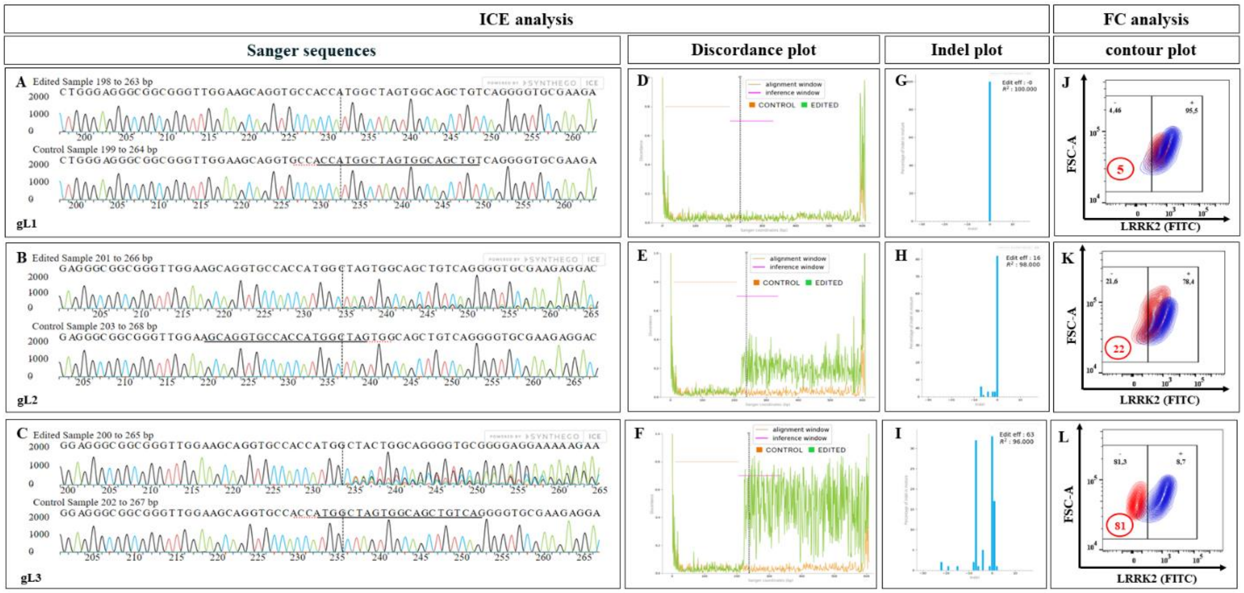

Supplementary Table S1. **Table from ICE analysis displaying ICE and KO scores as well as sgRNA sequences towards LRRK2.**

Knockout (KO) clones were sequenced by Sanger sequencing using primer LRRK2 seq to obtain knock-out efficiencies with the synthego ICE (Inference of CRISPR Edits) tool. Three crRNAs were designed for the LRRK2 locus at exon 1 in HEK-293 cells. The R^2 value (Pearson correlation coefficient) is a measure of how well the proposed indel distribution fits the Sanger sequence data of the edited sample. The maximum R^2 value is 1.0 and the sum of all individual contributions will be equal to the R^2 value. The R^2 value is critical for assessing the indel% and KO Score, as it sets the maximum value for these metrics. The higher the R2 value, the higher the confidence in the indel percentage and KO score. So, an R^2 value of 0.8 or more indicates a robust analysis, but R2 values below 0.8 should be considered with caution. All analyses obtained values over 0.8 knockout (KO) score is the proportion of sequences that are likely to result in functional protein knockout (frameshift mutations or indels of 21+ bp). The higher the KO score, the higher the percentage of sequences that result in a knockout of the target gene. The crRNA L3 showed the best efficiency (in **bold**).

Label	ICE	KO-Score	ICE d	R Squared	Mean Discord Before	Mean Discord After	Guide Sequences	LRRK2 seq Forward/Reverse primer (5'-3')
g L1	0	0	1	1	0,03220563	0,056255118	ACAGCTGCCACTAGCCATGG	ATAAACAGGCGGGCGTGGG/ TGCGGCTCCTTAAGAGTCCGG
gL2	16	15	26	0,98	0,032967001	0,208505456	GCAGGTGCCACCATGGCTAG	
**g L3**	63	**61**	69	0,96	0,031821972	0,493719012	CTGACAGCTGCCACTAGCCA	

Supplementary Table S2. Data of Normality and Variance Homogeneity tests.

Technique	Marker	Experimental group	Normality			Variance homogeneity		
			Shapiro-Wilk test			Levene test		
			P value	Passed yes/no (alpha=0.05)	P summary	P value	Passed yes/no (alpha=0.05)	P summary
Flow cytometry	Mitotracker	WT ROT 0	0.000	No	*	0.228	Yes	ns
		WT ROT 1	0.637	Yes	ns			
		WT ROT 5	0.637	Yes	ns			
		WT ROT 10	0.780	Yes	ns			
		WT ROT 50	0.780	Yes	ns			
		KO ROT 0	>0.999	Yes	ns			
		KO ROT 1	0.637	Yes	ns			
		KO ROT 5	0.637	Yes	ns			
		KO ROT 10	0.637	Yes	ns			
		KO ROT 50	0.726	Yes	ns			
	DCF	WT ROT 0	>0.999	Yes	ns	0.358	Yes	ns
		WT ROT 1	0.637	Yes	ns			
		WT ROT 5	0.637	Yes	ns			
		WT ROT 10	>0.999	Yes	ns			
		WT ROT 50	0.843	Yes	ns			
		KO ROT 0	0.637	Yes	ns			
		KO ROT 1	0.637	Yes	ns			
		KO ROT 5	>0.999	Yes	ns			
		KO ROT 10	0.637	Yes	ns			
		KO ROT 50	0.637	Yes	ns			
	DNA fragmentation	WT ROT 0	0.637	Yes	ns	0.846	Yes	ns
		WT ROT 1	>0.999	Yes	ns			
		WT ROT 5	0.637	Yes	ns			
		WT ROT 10	0.637	Yes	ns			
		WT ROT 50	0.637	Yes	ns			
		KO ROT 0	>0.999	Yes	ns			
		KO ROT 1	0.637	Yes	ns			
		KO ROT 5	>0.999	Yes	ns			
		KO ROT 10	0.637	Yes	ns			
		KO ROT 50	0.463	Yes	ns			
	Total-LRRK2	WT	0.726	Yes	ns	>0.999	Yes	ns
		KO	0.6369	Yes	ns			

	ox-DJ1	WT UNT	0.982	Yes	ns	0.242	Yes	ns
		WT ROT	0.788	Yes	ns			
		KO UNT	0.726	Yes	ns			
		KO ROT	0.759	Yes	ns			
	pS935-LRRK2	WT UNT	0.708	Yes	ns	0.269	Yes	ns
		WT ROT	0.575	Yes	ns			
		KO UNT	0.742	Yes	ns			
		KO ROT	0.700	Yes	ns			
	pS65-cJun	WT UNT	0.823	Yes	ns	0.236	Yes	ns
		WT ROT	0.201	Yes	ns			
		KO UNT	0.515	Yes	ns			
		KO ROT	0.228	Yes	ns			
	p53	WT UNT	0.614	Yes	ns	0.719	Yes	ns
		WT ROT	0.363	Yes	ns			
		KO UNT	0.962	Yes	ns			
		KO ROT	0.588	Yes	ns			
	PUMA	WT UNT	0.942	Yes	ns	0.210	Yes	ns
		WT ROT	>0.9999	Yes	ns			
		KO UNT	0.747	Yes	ns			
		KO ROT	0.4822	Yes	ns			
	CASP3	WT UNT	0,592	Yes	ns	0.011	No	*
		WT ROT	0481	Yes	ns			
		KO UNT	0.662	Yes	ns			
		KO ROT	>0.999	Yes	ns			
	PINK	WT UNT	0.803	Yes	ns	0.535	Yes	ns
		WT ROT	0.835	Yes	ns			
		KO UNT	0.696	Yes	ns			
		KO ROT	0.745	Yes	ns			
	PRKN	WT UNT	0.752	Yes	ns	0.839	Yes	ns
		WT ROT	0.122	Yes	ns			
		KO UNT	0.603	Yes	ns			
		KO ROT	0.985	Yes	ns			
	TOM20	WT UNT	0.804	Yes	ns	0.251	Yes	ns
		WT ROT	0.497	Yes	ns			
		KO UNT	0.921	Yes	ns			
		KO ROT	0.551	Yes	ns			
IF	TOM20/DRP1	WT UNT	0.477	Yes	ns	0.006	No	**
		WT ROT	0.945	Yes	ns			

KO UNT	0.946	Yes	ns
KO ROT	0.348	Yes	ns
